# Deep sequencing identification of miRNAs in pigeon ovaries illuminated with monochromatic light

**DOI:** 10.1186/s12864-018-4831-6

**Published:** 2018-06-08

**Authors:** Ying Wang, Hai-ming Yang, Wei Cao, Yang-bai Li, Zhi-yue Wang

**Affiliations:** College of Animal Science and Technology, Yangzhou University, Yangzhou, Jiangsu Province 225009 People’s Republic of China

**Keywords:** Pigeon, microRNA, Monochromatic light, Egg production

## Abstract

**Background:**

The use of light of different wavelengths has grown popular in the poultry industry. An optimum wavelength is believed to improve pigeon egg production, but little is known about the role of microRNAs (miRNAs) in the effects of monochromatic light on ovarian pigeon function. Herein, we harvested ovaries from pigeons reared under monochromatic light of different wavelength and performed deep sequencing on various tissues using an Illumina Solexa high-throughput instrument.

**Results:**

We obtained 66,148,548, 67,873,805, and 71,661,771 clean reads from ovaries of pigeons reared under red light (RL), blue light (BL), and white light (WL), respectively. We identified 1917 known miRNAs in nine libraries, of which 524 were novel. Three and five differentially expressed miRNAs were identified in BL vs. WL and RL vs. WL groups, respectively. Quantitative reverse transcription PCR was used to validate differentially expressed miRNAs (miR-200, miR-122, and miR-205b). In addition, 5824 target genes were annotated as differentially expressed miRNAs, most of which are involved in reproductive pathways including oestrogen signalling, cell cycle, and oocyte maturation. Notably, ovarian miR-205b expression was significantly negatively correlated with its target 11β-hydroxysteroid dehydrogenase type 1 (*HSD11B1)*.

**Conclusions:**

miRNA–mRNA network analysis suggests that miR-205b targeting of *HSD11B1* plays a key role in the effects of monochromatic light on pigeon egg production. These findings indicate that monochromatic light shortens the oviposition interval of pigeons, which may be useful for egg production and pigeon breeding.

**Electronic supplementary material:**

The online version of this article (10.1186/s12864-018-4831-6) contains supplementary material, which is available to authorized users.

## Background

Artificial illumination is widely used in the poultry industry, and both the optimum photoperiod and light wavelength have been investigated [1, 2]. We previously showed that red light (RL) supplementation increases the laying rate of pigeons and alters the expression of the circadian gene *BMAL1* [3, 4], and another investigation performed in experimental rooms reached the similar conclusion that RL promotes pigeon egg production while blue light (BL) has the opposite effect [5]. Additionally, our de novo transcriptome studies revealed that differentially expressed genes (DEGs) *E2F1*, *BMP15*, *HSD11B1*, and *Smad10* involved in monochromatic light affect pigeon reproduction (unpublished data). However, little is known about how post-transcriptional gene regulation affects egg production in pigeons.

MicroRNAs (miRNAs) are a class of endogenous small non-coding RNA that play vital roles in various processes by guiding the association between the RNA-induced silencing complex and target RNAs in reproductive tissues [6]. Functional miRNA targets are localised near the 3′ untranslated region (UTR) of protein-coding genes in relatively unstructured regions, which are occasionally in the 5’ UTR and within mRNA coding sequences [7–9]. Although a huge number of miRNAs have been identified in animals [10, 11], miRNAs in pigeons have not been reported to date. Xu et al. (2014) suggested that G-miR-143 is involved in ovarian function, including hormone secretion and reproduction processes [12]. Meanwhile, miR-202 is associated with oestrogen synthesis in chickens [13]. Thus, miRNAs are clearly important for ovarian activities in animals.

The White King pigeon (*Columba livia*) is an important commercial meat pigeon that has become popular in China. Monochromatic light can affect egg production in pigeons, but little is known about how it may influence pigeon reproduction. In this study, Illumina high-throughput sequencing was used to identify miRNAs involved in regulating pigeon follicular development under exposure to different monochromatic light-emitting diodes (LEDs). Combined with our previous ovary transcriptomic data, the results enhance our understanding of the mechanism by which monochromatic light shortens the oviposition interval in pigeons.

## Methods

### Ethics approval

This study was reviewed and approved by the Institutional Animal Care and Use Committee of the Department of Animal Science and Technology, Yangzhou University, and was performed in accordance with the Regulations for the Administration of Affairs Concerning Experimental Animals (China, 1988). All pigeon procedures were performed according to the Standards for the Administration of Experimental Practices (Jiangsu, China, 2008).

### Pigeon rearing and sample preparation

White King pigeons were raised in an isolated loft at the College of Animal Science and Technology, Yangzhou University (Yangzhou, China). A total of 108 paired birds were divided into red, blue, and white light groups, with three subgroups in each. The experiment lasted 6 months, and all pigeons were provided with food and water ad libitum. Birds were exposed to red (RL, 660 nm), blue (BL, 480 nm), or white/control (WL, 400 to 760 nm) LEDs (Shenzhen Hongda Technology Co., Ltd., Shenzhen, China) for 15 h each day (15 h light, 9 h dark). The light intensity was 15.20 ± 0.65 lx as measured with a TES-1336A light meter (TES Electrical Electronic Crop., Taipei, China). Egg production was recorded throughout the experimental period (120 days).

Nine female birds (three per group) of similar body weight (mean weight = 557.02 g) and similar physiological period (i.e., the day after the second egg was laid) were anesthetised with sodium pentobarbital at a dosage of 2.5 mg/100 g body weight. All efforts were made to minimise distress. Ovarian stromal tissues were immediately separated from follicles (large white, yellow, hierarchical, and post-ovulatory), flash-frozen in liquid nitrogen, and stored at − 80°C. The birds were released at the end of the experimentation.

### Small RNA library preparation and sequencing

Nine small RNA (sRNA) libraries were constructed from female pigeons raised under red (R1, R2, R3), blue (B1, B2, B3), or white (W1, W2, W3) light. Total RNA isolation was carried out from ovarian stromal tissues using TRIzol Reagent (Invitrogen, Carlsbad, CA, USA) according to the manufacturer’s instructions. Approximately 5 μg of total RNA from each test sample was used for sRNA sequencing. The quality of RNA samples was measured with an Agilent 2100 Bioanalyzer (Agilent Technologies, Santa Clara, CA, USA). sRNA fractions were ligated to 3′ and 5′ adaptors, and adaptor-ligated sRNAs were subjected to quantitative reverse transcription PCR (RT-PCR) with 15 cycles of amplification. PCR products were purified using 4% agarose gels and used for sequencing on the Illumina HiSeq 4000 platform at Shanghai Oebiotech Co., Ltd. (Illumina, San Diego, CA, USA; Shanghai Oebiotech Co., Ltd., China).

### Bioinformatics analysis

Clean data were obtained by removing adapter sequences, cleaning low-quality tags, and filtering adaptor-ligated contaminants and short read tags (< 18 nt). Reads were then aligned with the *Columba livia* genome using the Short Oligonucleotide Alignment Program (SOAP) [13]. Clean reads were compared against sRNAs (rRNAs, tRNAs, snRNAs, snoRNAs, and miRNA) deposited in GenBank and Rfam (http://www.sanger.ac.uk/resources/databases/rfam.html) databases to annotate sRNA sequences. Reads were aligned against known miRNA precursors and mature miRNAs deposited in miRBase 20.0 to identify conserved miRNAs. The hairpin structure that is characteristic of miRNA precursors can be used to predict novel miRNAs. Star sequences were categorised using miRDeep2, and secondary structures were identified with the RNAfold tool [14].

miRNA expression levels were compared between pairs of groups. Firstly, data were normalised to obtain transcripts per million values using the following formula normalised expression = (actual miRNA count / total count of clean reads) × 1,000,000. Fold-change and *p*-values were then calculated from normalised expression values. Differential miRNA expression in the two groups was analysed with DESeq (http://bioconductor.org/packages/release/bioc/html/DESeq.html).

### Target gene prediction

Potential target sites of miRNA candidates were identified by aligning miRNA sequences with the integrated pigeon transcriptome. To gain further insight into the functions and classifications of the identified miRNAs targets, we performed Gene Ontology (GO) term and Kyoto Encyclopaedia of Genes and Genomes (KEGG) pathway annotation of predicted miRNA targets using the DAVID gene annotation tool (http://david.abcc.ncifcrf.gov/). We used KOBAS software to test the statistical enrichment of potential target mRNAs in GO and KEGG pathways. Based on previously identified DEGs between RL vs. WL and BL vs. WL groups using transcriptome sequencing (available at the NCBI SRA database under accession code PRJNA289165), we selected potential target mRNAs related to egg production. A regulatory network of differentially expressed mRNAs (DEMs) and potential target mRNAs was established using Cytoscape software, and genes with false discovery rates ≤0.05 were considered significantly enriched.

### RT-qPCR for mature miRNAs

RT-qPCR was used to validate differentially expressed miRNAs. Samples were isolated from pigeon ovarian stromal tissues using an miRcute miRNA Isolation Kit (DP501; TIANGEN, Beijing, China), and 3 mL of sRNA was subjected to RT using the miRcute miRNA First-Strand cDNA Synthesis kit. Poly(A) management and RT-PCR conditions were as described in the manufacturer’s recommendations. SYBR Green RT-PCR assays using the miRcute miRNA qPCR detection kit (FP401, TIANGEN) were conducted to measure miRNA expression according to the manufacturer’s protocol (primer sequences are listed in Additional file 1). The reference gene U6 served as an internal reference. Each sample was analysed three times, and relative miRNA expression was calculated using the 2^-ΔΔCT^ method [15].

### Cli-miR-205b target prediction and *HSD11B1* luciferase reporter assay

miRanda software was used to predict target genes of cli-miR-205b by searching the 3’ UTR sequences of genes identified from RNA-seq. Combining the results of target genes of differentially expressed microRNAs and DEGs from RNA-seq, *HSD11B1* was selected as a putative target gene of cli-miR-205b. Expression levels of cli-miR-205b and *HSD11B1* in ovaries were then examined with RT-qPCR.

The 3’ UTR regions of *HSD11B1* fragments were amplified from genomic DNA and the pmirGLO vector (Promega, Madison, WI, USA) with *Nhe*I and *Xho*I restriction enzyme sites at the 3’ UTR region of the luciferase gene to construct the luciferase reporter plasmid pmirGLO-Wt/pmirGLO-Mut. Primer sequences used to amplify 3’ UTR regions are listed in Additional file 1. Mutagenesis of the miR-205b target site in the *HSD11B1* 3’ UTR was carried out using a QuikChange site-directed mutagenesis kit (Promega) with pmirGLO-Wt as the template, resulting in the mutant reporter plasmid pmirGLO-Mut. For the luciferase reporter assay, 293 T cells were seeded in 24 well plates and transfected with 50 nM mimics of miR-205b or scrambled microRNA (mimics-NC) and 1 μg luciferase reporter plasmid pmirGLO-Wt/pmirGLO-Mut using an X-tremegene HP (Roche, Basel, Switzerland). At 48 h after transfection, cells were harvested, and luciferase activity was measured using the dual-luciferase reporter assay (Promega) [16].

### Statistical analysis

Data are expressed as mean ± standard deviation, and one-way analyses of variance was performed with SPSS 13.0 software (SPSS Inc., Chicago, IL, USA). The statistical significance of differences among the various groups was evaluated by least significant difference post hoc multiple comparisons tests, and *p* < 0.05 was considered statistically significant.

## Results

### Sequence analysis of short RNAs

**Fig. 1:**
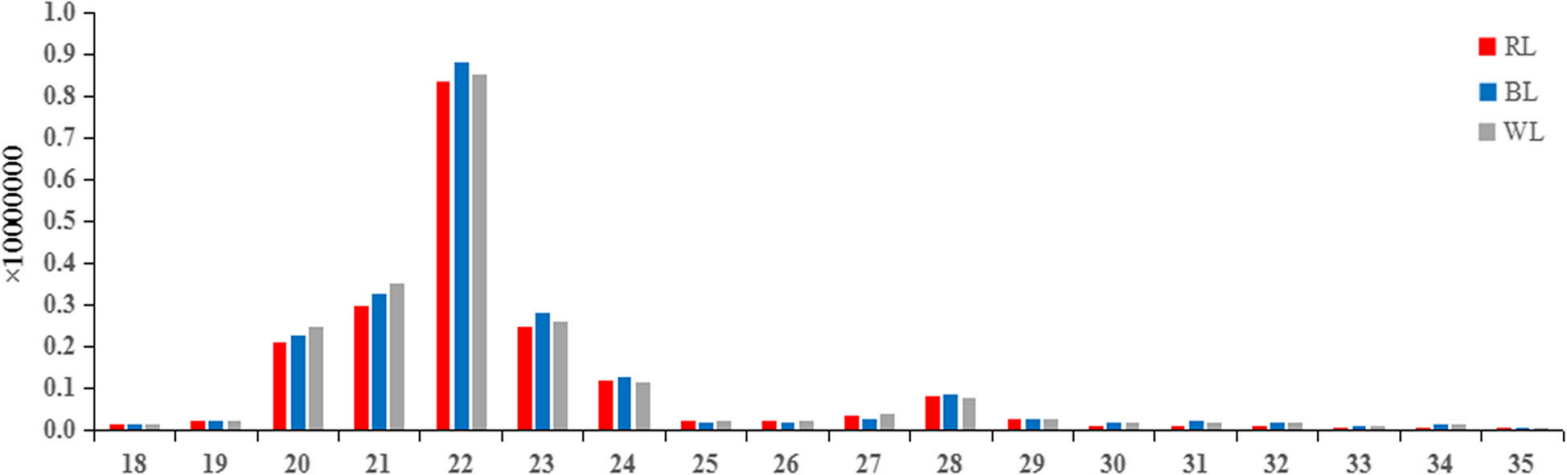
Length distribution of the unique sRNA sequences in the three sRNA libraries

**Fig. 2:**
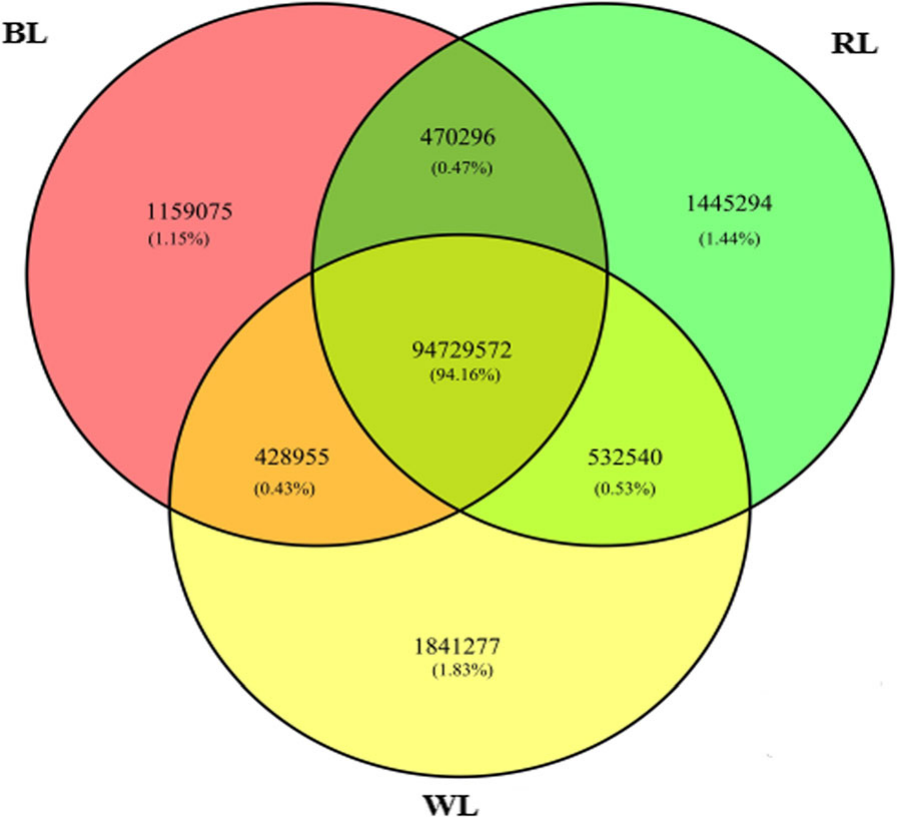
Venn diagram of total sRNA reads in the three libraries. The overlapping sector shows common sequences, the other sectors show the unique sequences in each

**Fig. 3:**
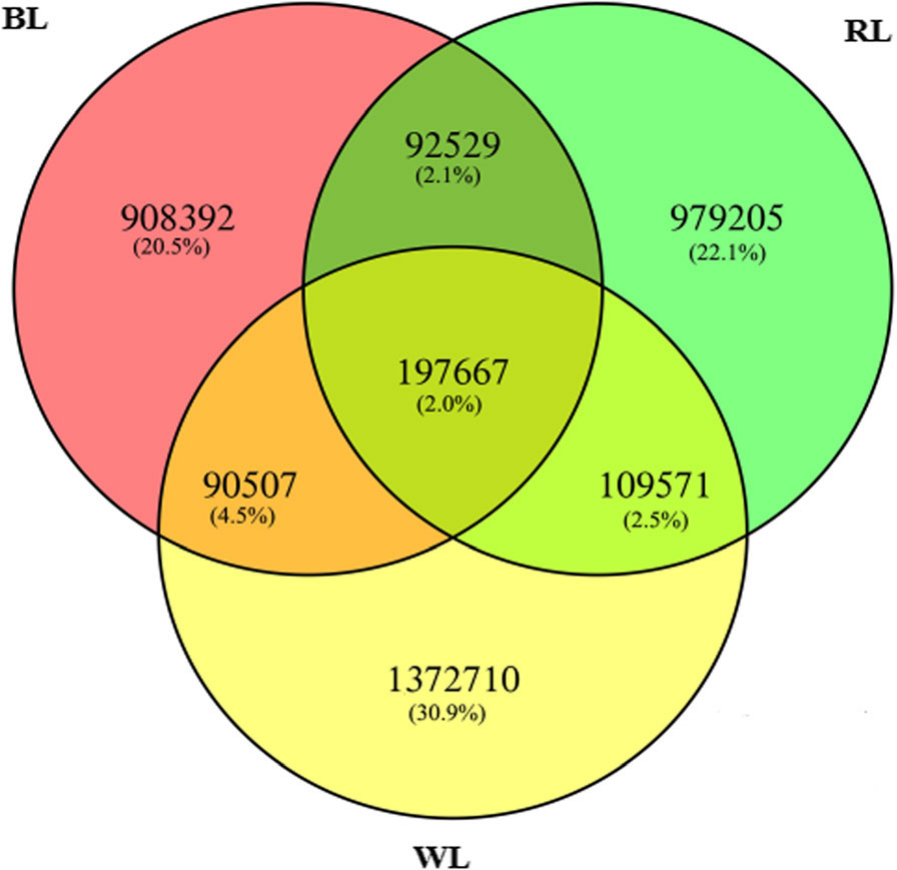
Venn diagram of unique sRNA reads in the three libraries. The overlapping sector shows common sequences, the other sectors show the respective specific sequences in each

**Fig. 4:**
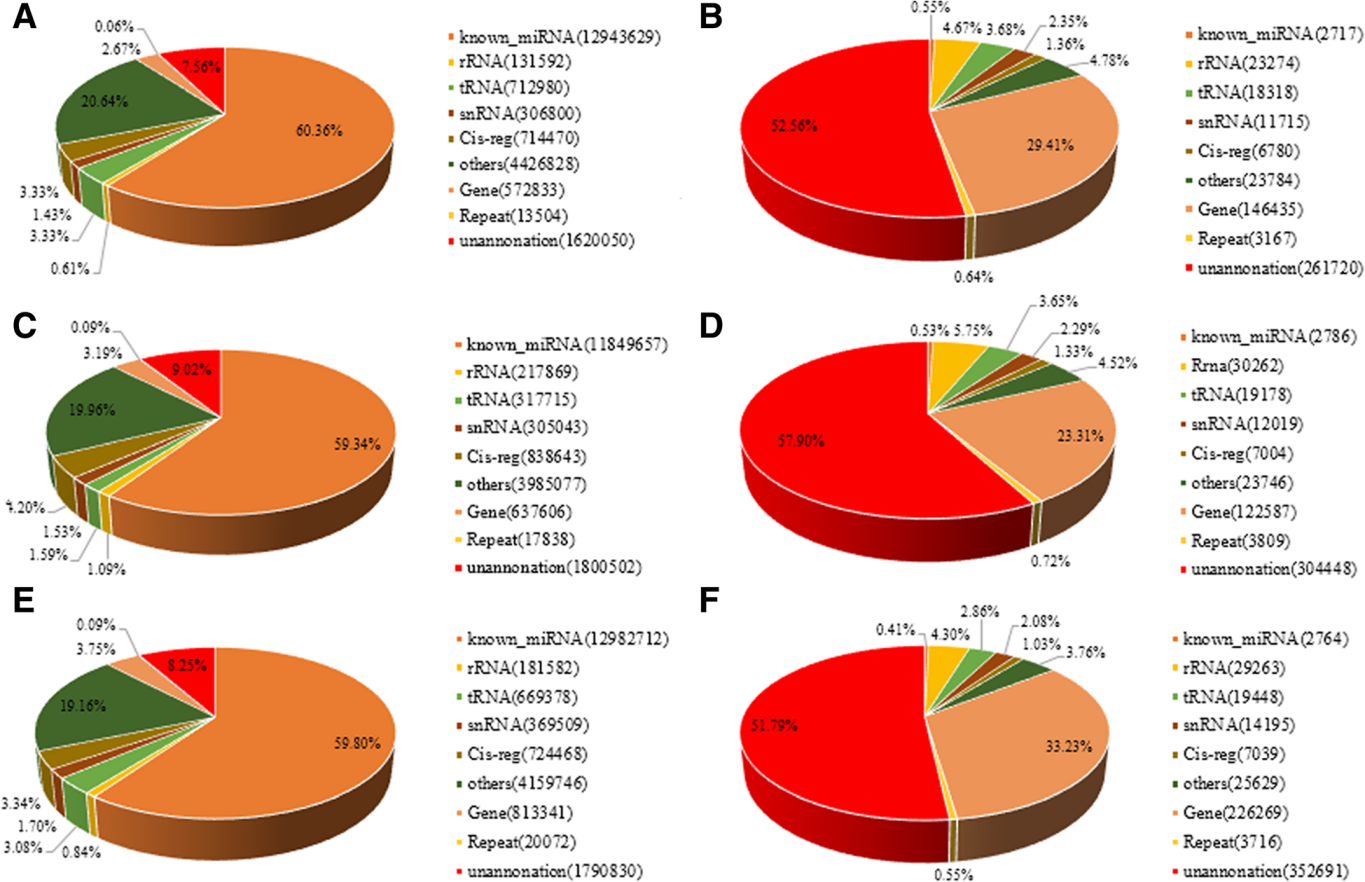
Composition of sRNA categories in the three libraries. **a** Total number of reads in the BL library. **b** Total number of unique sequences in the BL library. **c** Total number of reads in the RL library. **d** Total number of unique sequences in the RL library. **e** Total number of reads in the WL library. **f** Total number of unique sequences in the WL library

Nine sRNA libraries were built from RL (*n* = 3), BL (*n* = 3) and WL (*n* = 3) groups, respectively. Clean reads ranged from 18 to 41 nt, and the size distribution of clean reads is shown in Fig. 1. Most were 20−24 nt in length, which is consistent with the typical size range for sRNAs generated by Dicer. We obtained 66,148,548, 67,873,805, and 71,661,771 clean reads, representing 2,256,228 (RL), 2,543,975 (BL), and 33,39,478 (WL) unique sequences in the three respective libraries. Alignment against the *Columba livia* genome using the SOAP program showed that 62.74% (RL), 66.52% (BL), and 69.47% (WL) of sequences mapped onto reference sequences, and the remainder were aligned with other types of RNA. Comparison of total and unique sRNA reads suggested that most of the total sRNA reads were common among libraries, whereas only 2.0% of total sRNA common sequences were common to all three libraries, and most unique sRNA reads were library-specific (Figs. 2 and 3). All clean reads were annotated and classified by aligning against the Rfam 10.1, miRBase 20.0, and GenBank databases. Conserved miRNAs accounted for 60.36, 59.34, and 59.80% of total clean reads in BL, RL, and WL sRNA libraries, respectively (Fig. 4). Furthermore, miRNAs accounted for 0.55, 0.53, and 0.41% of unique reads in the three respective libraries. These results confirmed the success of the Illumina Hiseq sequencing procedure.

### Differentially expressed miRNAs in different monochromatic light groups

Using the miRBase database, we identified 1917 known miRNAs in nine libraries, and found three and five differentially expressed miRNAs in BL vs. WL and RL vs. WL groups, respectively (Additional file 2). In addition, 524 novel miRNAs were identified, with 10 and 15 differentially expressed novel miRNAs in BL vs. WL and RL vs. WL groups, respectively. The identified pre-miRNAs displayed a typical stem-loop structure and calculated free energy ranging from − 101.07 to − 30.01 kcal/mol (Additional file 3). miRNA precursor folding structures are shown in Additional file 4.

### RT-qPCR validation of pigeon miRNAs

**Fig. 5:**
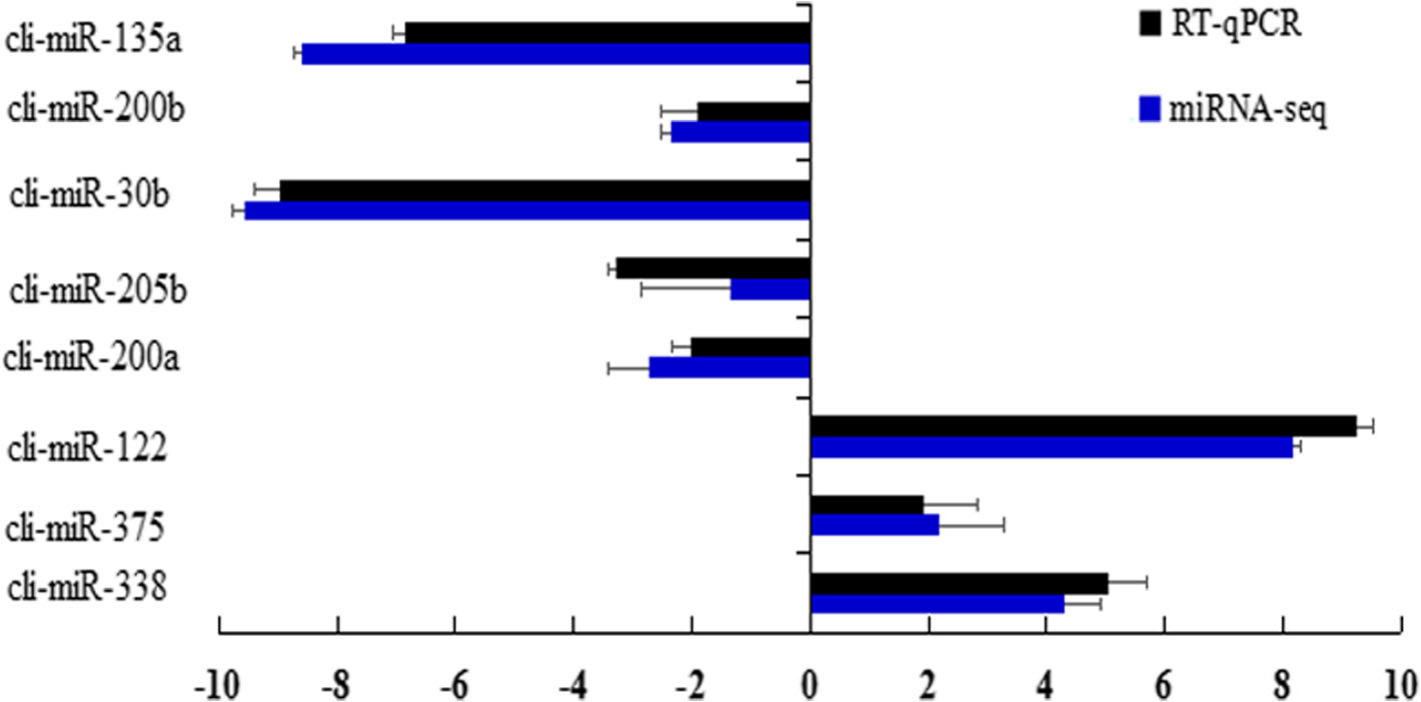
High-through sequencing and RT-qPCR results

RT-qPCR was used to measure the expression of differentially expressed miRNAs to validate the reliability of microRNA sequencing. Nine microRNAs were selected. Consistent with the high-throughput sequencing results, miR-30b, miR-135a, and miR-200b were up-regulated, while miR-338, miR-205b, miR-200a, miR-122, and miR-375 were down-regulated (Fig. 5).

### miRNA target gene prediction, GO enrichment, and KEGG pathway analysis

**Fig. 6:**
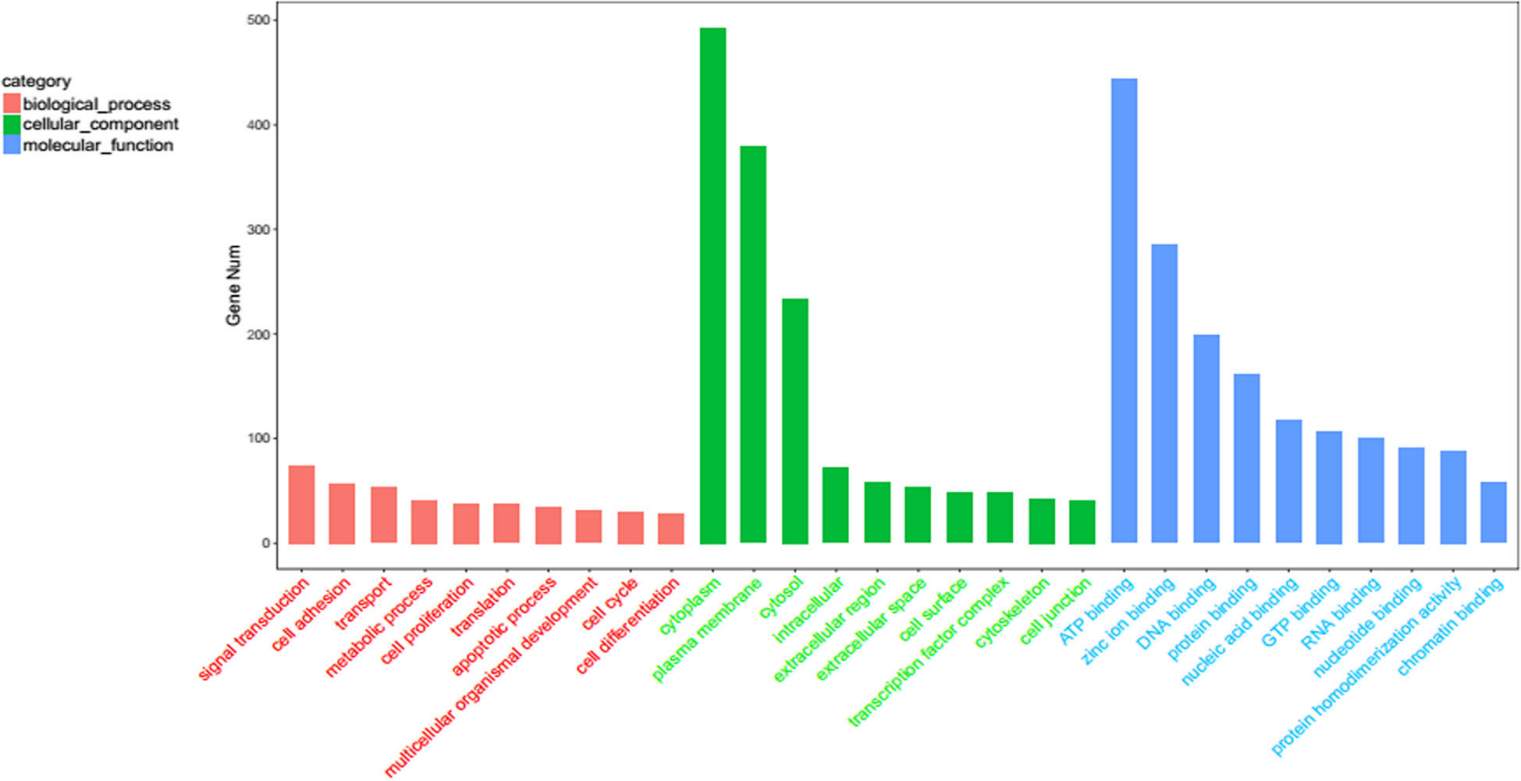
Gene ontology classification for potential target genes of differentially expressed miRNAs. Target genes associated with biological processes, cellular component, and molecular function

To further understand the functions of differentially expressed miRNAs in pigeons exposed to monochromatic light of different wavelengths, miRNA target gene prediction was performed based on our transcriptome results. GO enrichment analysis was performed on target gene candidates of the identified differentially expressed miRNAs. A total of 682 genes were identified in the cellular component category, the biological process category contained 4026 genes involved in signal transduction, cell proliferation, translation, and cell cycle, and 1634 genes in the molecular function category were related to ATP, GTP, and chromatin binding (Fig. 6).

Target genes of differential miRNAs were classified to identify pathways according to KEGG functional annotation (Additional file 5). A total of 5824 target genes were annotated for differentially expressed miRNAs, mostly involved in signal transduction and cellular metabolism, but also others related to reproductive pathways including oestrogen signalling, cell cycle, and oocyte maturation.

We further filtered key target genes related to the effects of monochromatic light based on our previously identified DEGs and functional enrichment. A regulatory network between differentially expressed miRNAs and key target genes was also constructed and analysed (Additional file 6). Based on this, we speculated that *HSD11B1* was the target of down-regulated cli-miR-205b.

### Validation of miRNA–mRNA interactions using cli-miR-205b mimics

**Fig. 7:**
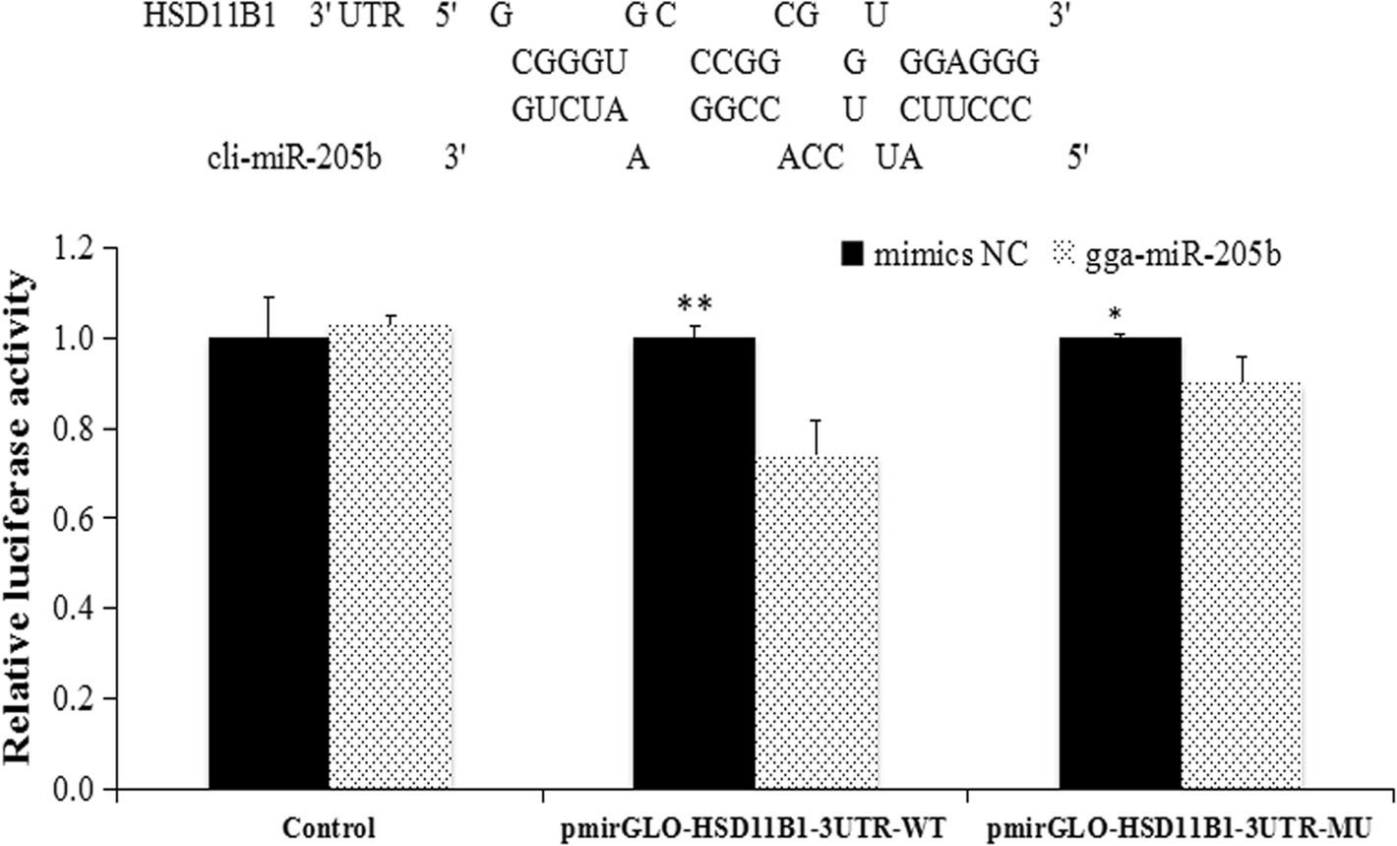
*HSD11B1* regulation by cli-miR-205b. Predicted cli-miR-205b binding sites at distinct positions in *HSD11B1*; nucleotides in the cli-miR-205b seed region. Luciferase activity in 293 T cells transfected with miRNA mimics and plasmids carrying the 3′ UTR of *HSD11B1*. NC miRNA = negative control miRNA. **represents *P* < 0.01, *represents *P* < 0.05

Firstly, we measured the relative expression of cli-miR-205b and its target *HSD11B1* using RT-qPCR (Additional file 7). The findings were consistent with the miRNA-seq and RNA-seq results; cli-miR-205b was more highly expressed in the BL group than the RL group (*p* < 0.05), while *HSD11B1* was highly expressed in the RL group compared with the BL group (*p* < 0.05). This suggests that cli-miR-205b inhibited *HSD11B1* expression. Secondly, we predicted that the binding site for cli-miR-205b was in the 3’ UTR, and the luciferase reporter gene system was used to validate the interaction. The 3’ UTR of *HSD11B1* was cloned into luciferase reporter plasmids to test cli-miR-205b function in vitro. The results showed that the cli-miR-205b mimic induced a significant reduction in the relative luciferase activity of the *HSD11B1* plasmids (Fig. 7, *p* < 0.001) compared with negative control miRNA and the no-insert control. Furthermore, it also significantly affected pmirGLO-Mut activity (*p* < 0.05). These results indicate that cli-miR-205b probably down-regulates the expression of HSD11B1 by binding to the 3’ UTR, and this appears to play a key role in the effects of monochromatic light on pigeon egg production.

## Discussion

Pigeons engage in close pair bonding, produce two eggs in a laying period, and squabs are fed with crop milk regurgitated from both parents [17, 18]. The breeding cycle of pigeons in the wild is nearly 2 months [19], and Khargharia et al. (2003) reported a mean clutch interval of 47.44 days [20]. These parameters could be improved meet increasing consumer demand. We previously reported on the effectiveness of monochromatic light supplementation on a pigeon farm, and a monochromatic light regime in experimental rooms (15 L:9D) altered egg production. Both the present study and Wang et al. (2015) showed that RL and BL increase and decrease egg production, respectively (Wang et al., 2015). On this basis, we further analysed DEG patterns, important pathways, and miRNAs in tissues from pigeons raised under different monochromatic light wavelengths using microarray and Solexa sequencing technologies (data available at the NCBI SRA database under SRA Project accession number SRP124987, and BioProject accession number PRJNA418062).

Carletti and Christenson (2014) concluded that miRNAs are key posttranscriptional regulators that modulate translation or degradation of their target mRNAs, and these processes play important roles in ovaries and other female reproductive tissues [6, 21, 22]. Monochromatic light exposure impacts pigeon egg production and has a significant effect on mRNA expression, and our results showed that miRNAs participate in gene regulation. Because miRNA candidate identification was based on known *Columba livia* genome sequences (the matching ratio between rock pigeon and White King pigeon was low), there may be a few sequence differences.

Numerous studies have linked the differentially expressed miRNAs identified in the present work to various reproductive pathways. Sirotkin et al. (2009a) concluded that miR-30a-3p, miR-135, and miR-122 inhibit the release of oestradiol, testosterone, and progesterone [23, 24], while Mattes and colleagues (2007) confirmed that the murine ovarian-specific miRNA miR-30 is involved in ovarian steroid hormone release [25]. Hasuwa et al. (2013) suggested that miR-200b suppresses the expression of the transcriptional repressor *ZEB1*, which is directly up-regulated by progesterone/PR action at the *ZEB1* promoter [26, 27]. Menon et al. (2013) found that increased levels of luteinising hormone receptor (LHR) mRNA binding protein induces down-regulation of *LHR* mRNA expression, and this is mediated by miR-122 [28, 29]. Estradiol exposure reduces miR-338-3p levels [30], and Wang et al. (2016) found that miR-338 and miR-200a are negatively correlated with *CYP17A2*, which helps regulate steroid hormone biosynthesis [31]. miR-375 is involved in chicken ovary maturation, and its overexpression suppresses glucose-induced insulin secretion [32, 33].

Letzen et al. (2010) reported that the miR-205 family is highly expressed in undifferentiated embryonic stem cell and early neural progenitor stages, and is down-regulated during the glial restricted and early oligodendrocyte progenitor transitions [34]. Xiao et al. (2014) proposed that up-regulated miR-205 in MI pig oocytes is involved in insulin-like growth factor 1/brain-derived neurotropic factor-induced oocyte maturation [35, 36]. miR-205 regulates animal reproduction via its effects on multiple target genes. Our RNA-seq results identified *HSD11B1*, which is a known target gene of miR-205b. *HSD11B1* catalyses the interconversion of inactive cortisone and active cortisol, which is important for conceptus elongation and implantation during peri-implantation [37]. 11βHSD catalyses the conversion between active and inactive glucocorticoids; during follicular maturation, glucocorticoid suppresses differentiation by downregulating P450 aromatase and LHR expression in granulosa cells, and 11HSD1 expression is developmentally regulated in maturing bovine follicles [38, 39]. In oestrus-synchronised control animals, moderate 11βHSD1 expression was observed in granulosa cells, with an increase in the transition from primary to atretic follicles [40]. In future studies, we will perform miR-205b overexpression and RNA interference studies in pigeon ovarian granulosa cells to examine the effects on the expression of *HSD11B1* and other important genes related to steroid hormone biosynthesis pathways, as well as changes in steroid hormone concentrations.

## Conclusion

In conclusion, we performed the first sRNA-seq analysis of pigeon miRNAs and identified miR-122, miR-200, and miR-205b as candidate miRNAs involved in the effects of monochromatic light on pigeon reproduction. Network analysis suggests that *HSD11B1* is a potential target gene of miR-205b and likely plays a key regulatory role in steroid hormone biosynthesis, granular cell development, and steroid hormone biosynthesis pathways. These findings provide insight into the mechanism by which monochromatic light shortens the oviposition interval of pigeons, which may prove useful for egg production and pigeon breeding.

## Additional files

Additional file 1: Primers used in the experiments. (DOC 29 kb)

Additional file 2: Differential expressions of known miRNAs in ovary identified in BL vs. WL and RL vs. WL. (DOC 25 kb)

Additional file 3: Characteristics of novel miRNA candidates. (XLS 337 kb)

Additional file 4: Partial secondary structures of novel microRNAs. (TIF 287 kb)

Additional file 5: KEGG pathway annotations for the target genes of differentially expressed miRNAs. (XLS 2500 kb)

Additional file 6: Network analysis of differentially expressed miRNAs interacting with potential target genes related to the effect of monochromatic light on pigeon egg production. (TIF 1820 kb)

Additional file 7: Expression levels of miR-205b and target HSD11B1 in pigeon ovary under different monochromatic lights. (TIF 458 kb)

## Abbreviations

BL: Blue light
DEGs: Differential expression genes
HSD11B1: 11β-hydroxysteroid dehydrogenase type 1
LEDs: Light-emitting diodes
RL: Red light
RT-qPCR: Quantitative reverse transcription polymerase chain reaction
WL: White light
