# Supplementary material for: Deep sequencing identification of miRNAs in pigeon ovaries illuminated with monochromatic light
Source: BMC Genomics. 2018 Jun 8;19:446. doi: 10.1186/s12864-018-4831-6 (PMC5994017; doi:10.1186/s12864-018-4831-6)
Supplement: Supplementary file 1 — Primers used in the experiments. (DOC 29 kb) [file 12864_2018_4831_MOESM1_ESM.doc]

Primers and RNAs used in this study

| Category | Primers and RNA name | Forward primer (5'-3') |
| --- | --- | --- |
| Mature miRNA | cli-miR-205b | | CCUUCAUUCCACCGGAAUCUG | | --- | |
|  | | cli-miR-30b | | --- | | UGUAAACAUCCUACACUCAGC |
|  | cli-miR-200a | UAACACUGUCUGGUAACGAUG |
|  | cli-miR-122 | GAGUGUGACAAUGGUGUUUGU |
|  | cli-miR-375 | UUGUUCGTTCGGCUCGCGUU |
|  | cli-miR-135a | AUGUAGGGCGAAAAGCCAUG |
|  | cli-miR-200b | CAUCUUACUGGGCAGCAUUG |
|  | cli-miR-338 | | AACACUAUCCUGAUGCUGUCA | | --- | |
|  | U6 | CAAGGAUGACACGCAAAUUCG |
| pmirGLO-HSD11B1-3UTR-WT | F | AAACGAGCTCGCTAGCAGGGTTTGGAACGAACGGCG |
|  | R | CGACTCTAGACTCGAGGGTTAGTAAATTTTATTTAATAATTAG |
| pmirGLO-HSD11B1-3UTR-MU | F | AAACGAGCTCGCAGGGTTTGGAACGAACGGTCCCTGCGTGCGCG |
|  | Mutate primers | CGAACGGTCCCTGCGTGCGCGCCAGCCTTTCGCCGGTGCAACAGCGTC |
|  | R | CGACTCTAGACTCGAGGGTTAGTAAATTTTATTTAATAATTAG |
| HSD11B1 | F | AAGGATTTGCATTGCCGCTTC |
|  | R | TTTGAAATTCTCCCTCGCAGA |
