# Supplementary material for: Deep sequencing identification of miRNAs in pigeon ovaries illuminated with monochromatic light
Source: BMC Genomics. 2018 Jun 8;19:446. doi: 10.1186/s12864-018-4831-6 (PMC5994017; doi:10.1186/s12864-018-4831-6)
Supplement: Supplementary file 2 — Differential expressions of known miRNAs in ovary identified in BL vs. WL and RL vs. WL. (DOC 25 kb) [file 12864_2018_4831_MOESM2_ESM.doc]

**Table S2.** Differential expression of known miRNAs in ovary identified in BL vs. WL and RL vs. WL.

| **miR-Name** | **Mature sequence (5'→3')** | **WL** | **BL** | **Fold-Change (log2BL/WL)** | **Expression Level** |
| --- | --- | --- | --- | --- | --- |
| cli-miR-135a | auguagggcgaaaagccauggg | 0.291899505 | 5.743323452 | 4.298342144 | Up |
| cli-miR-200b | caucuuacuuacugggcagcauugg | 6.271297103 | 28.56769235 | 2.187548729 | Up |
| cli-miR-338 | aacacuauccugaugcuguca | 3.91355128 | 0.01 | -8.612334539 | Down |
| **miR-Name** | **Mature sequence (5'→3')** | **WL** | **RL** | **Fold-Change (log2RL/WL)** | **Expression Level** |
| cli-miR-205b | cccuucauuccaccggaaucugu | 995.0297989 | 151.4210408 | -2.716174042 | Down |
| cli-miR-30b | uguaaacauccuacacucagc | 0.01 | 2.889409748 | 8.174630997 | Up |
| cli-miR-200a | uaacacugucugguaacgaug | 58.51088239 | 22.74554522 | -1.363120958 | Down |
| cli-miR-122 | uggagugugacaaugguguuugu | 7.617369117 | 0.01 | -9.573148996 | Down |
| cli-miR-205b | cccuucauuccaccggaaucug | 577.4436774 | 99.35750561 | -2.538979376 | Down |
| cli-miR-375 | uuuguucguucggcucgcguu | 237.2059554 | 46.08896459 | -2.363646969 | Down |
