# Supplementary figures and images for: Deep sequencing identification of miRNAs in pigeon ovaries illuminated with monochromatic light
Source: BMC Genomics. 2018 Jun 8;19:446. doi: 10.1186/s12864-018-4831-6 (PMC5994017; doi:10.1186/s12864-018-4831-6)

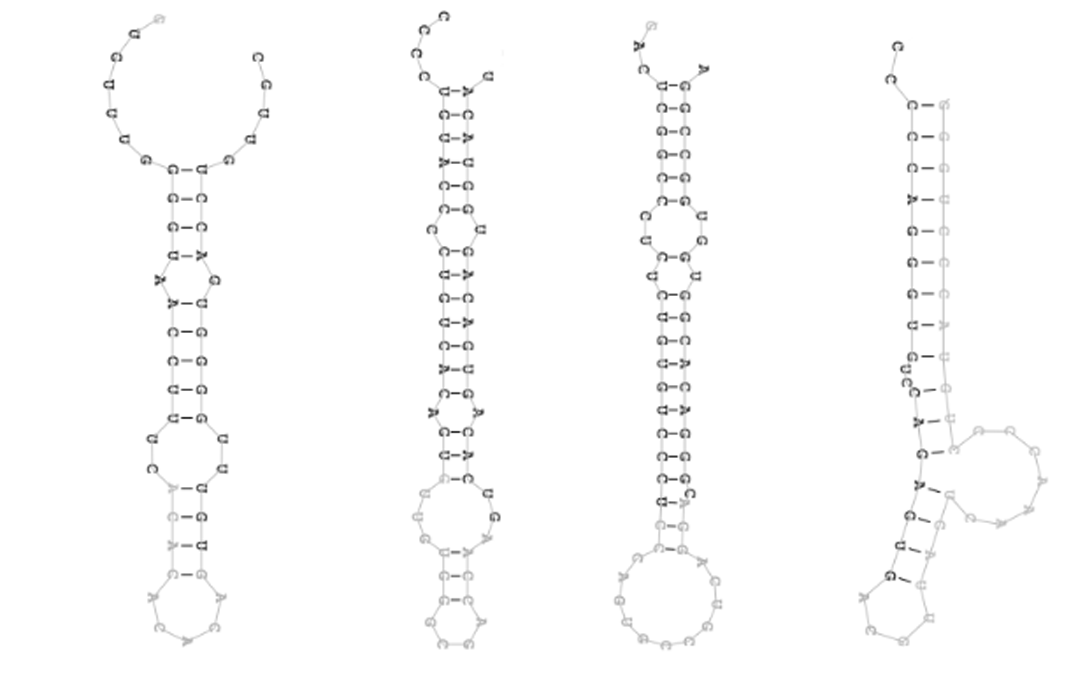

Supplement: Supplementary file 4 — Partial secondary structures of novel microRNAs. (TIF 287 kb) [file 12864_2018_4831_MOESM4_ESM.tif]

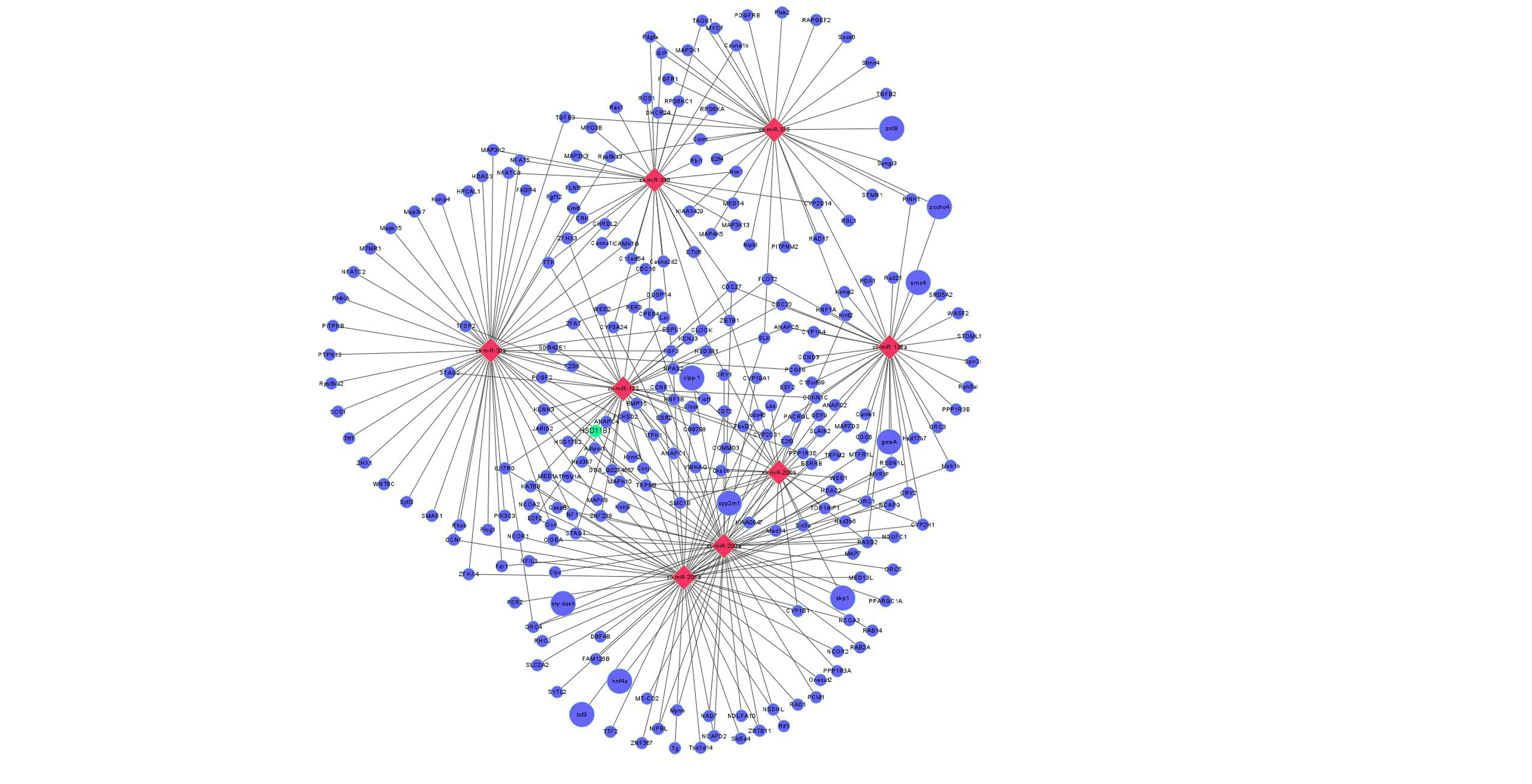

Supplement: Supplementary file 6 — Network analysis of differentially expressed miRNAs interacting with potential target genes related to the effect of monochromatic light on pigeon egg production. (TIF 1820 kb) [file 12864_2018_4831_MOESM6_ESM.tif]

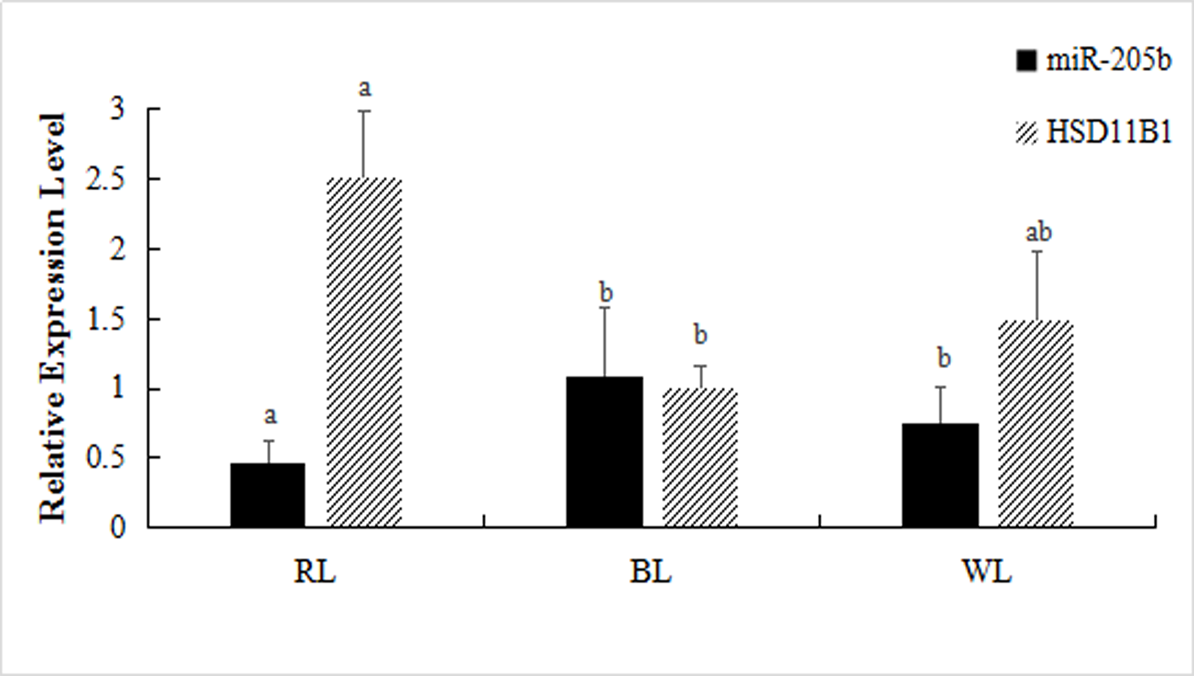

Supplement: Supplementary file 7 — Expression levels of miR-205b and target HSD11B1 in pigeon ovary under different monochromatic lights. (TIF 458 kb) [file 12864_2018_4831_MOESM7_ESM.tif]
